# Social Media for Knowledge Acquisition and Dissemination: The Impact of the COVID-19 Pandemic on Collaborative Learning Driven Social Media Adoption

**DOI:** 10.3389/fpsyg.2021.648253

**Published:** 2021-05-31

**Authors:** Muhammad Naeem Khan, Muhammad Azeem Ashraf, Donald Seinen, Kashif Ullah Khan, Rizwan Ahmed Laar

**Affiliations:** ^1^School of Social and Behavioral Science, Nanjing University, Nanjing, China; ^2^Research Institute of Educational Science, Hunan University, Changsha, China; ^3^School of Information Management, Nanjing University, Nanjing, China; ^4^School of Management Sciences, Ghulam Ishaq Khan Institute of Engineering Sciences and Technology, Swabi, Pakistan; ^5^School of Sports Science and Physical Education, Nanjing Normal University, Nanjing, China

**Keywords:** collaborative learning, COVID-19, electronic-learning, social networking, student community, TAM

## Abstract

During the COVID-19 outbreak, educational institutions were closed, and students worldwide were confined to their homes. In an educational environment, students depend on collaborative learning (CL) to improve their learning performance. This study aimed to increase the understanding of social media adoption among students during the COVID-19 pandemic for the purpose of CL. Social media provides a learning platform that enables students to easily communicate with their peers and subject specialists, and is conducive to students' CL. This study addresses the key concept of CL during the COVID-19 pandemic by assessing social media use among students in higher education. The relationship between social media use and students' performance is crucial to understanding the role of social media during a pandemic. This study is based on constructivism theory and the technology acceptance model. Structural equation modeling was used to analyze the conceptual model using SmartPLS. The research findings indicate that social media plays an important role during the pandemic because it provides opportunities for students to enhance CL under the aforementioned situations. This study makes noteworthy theoretical and practical contributions.

## Introduction

The onset of the COVID-19 outbreak is a historically unparalleled adverse occurrence (Venkatesh, 2020) with unprecedented lockdowns imposed across nations Pandemics are large outbreaks of an infectious disease over a wide geographical area and can result in widespread morbidity and mortality (Madhav et al., 2017). Various governments have imposed restrictions on citizens' movements, canceled social activities, and advised people to stay at home to prevent the spread of COVID-19 (Laato et al., 2020). The grim fact is that thousands of people have experienced and will continue to experience the negative impacts of COVID-19, including its induced constraints (Dwivedi et al., 2020).

COVID-19 has disrupted many aspects of life, such as the medical system, economy, and education (Li et al., 2020). Educational institutions saw forced closures, and students were required to remain at home. This has led to procedural changes in the day-to-day operations of academic institutions. Because of the pandemic, digital advances have been made in the global higher education sector (Dwivedi et al., 2020). Colleges and universities have canceled classes, and administrators have struggled to convert courses into an online format in a matter of days or weeks (McMurtrie, 2020). To efficiently deliver this online content, educational institutions have created official websites and applications to enable students to continue their education. Several academic institutions have implemented “e-learning,” a web-based learning ecosystem for the dissemination of information and communication to support instructors in their transition to online learning (McMurtrie, 2020).

During the pandemic, people tended to spend more time on social media (SM) as they practice social distancing. SM sites saw an increase of 61% in web traffic compared to the typical rates during the first 3 months of the COVID-19 outbreak. In the past, students reported that SM adoption is, in part, motivated by the need to contact family, teachers, classmates, colleagues, and friends (Holmes, 2020). SM is a medium of communication that enables teachers and students to communicate through numerous online learning applications while adhering to social distancing regulations (Vordos et al., 2020). Furthermore, during COVID-19, the role of SM for educational purposes has become more significant, as it enhances connectivity and brings collaborative opportunities to people who are now beginning to use SM (Islam et al., 2020). SM tools enable teachers, students, and academic institutions to change their teaching or learning methods in a bid to overcome COVID-19 induced restrictions. With the growth of social networks and the increased online presence of many academic institutions, students enjoy live streaming services through SM (e.g., Instagram, Facebook) whereby they participate in regular discussions on trending topics and keep in touch with peers or instructors via online forums (Abi-Rafeh et al., 2019; Abi-Rafeh and Azzi, 2020). Regarding teaching and learning purposes in the context of assessing education sustainability, researchers have examined the incorporation of learning via SM in the higher education curriculum (Alamri et al., 2020).

**Figure 1:**
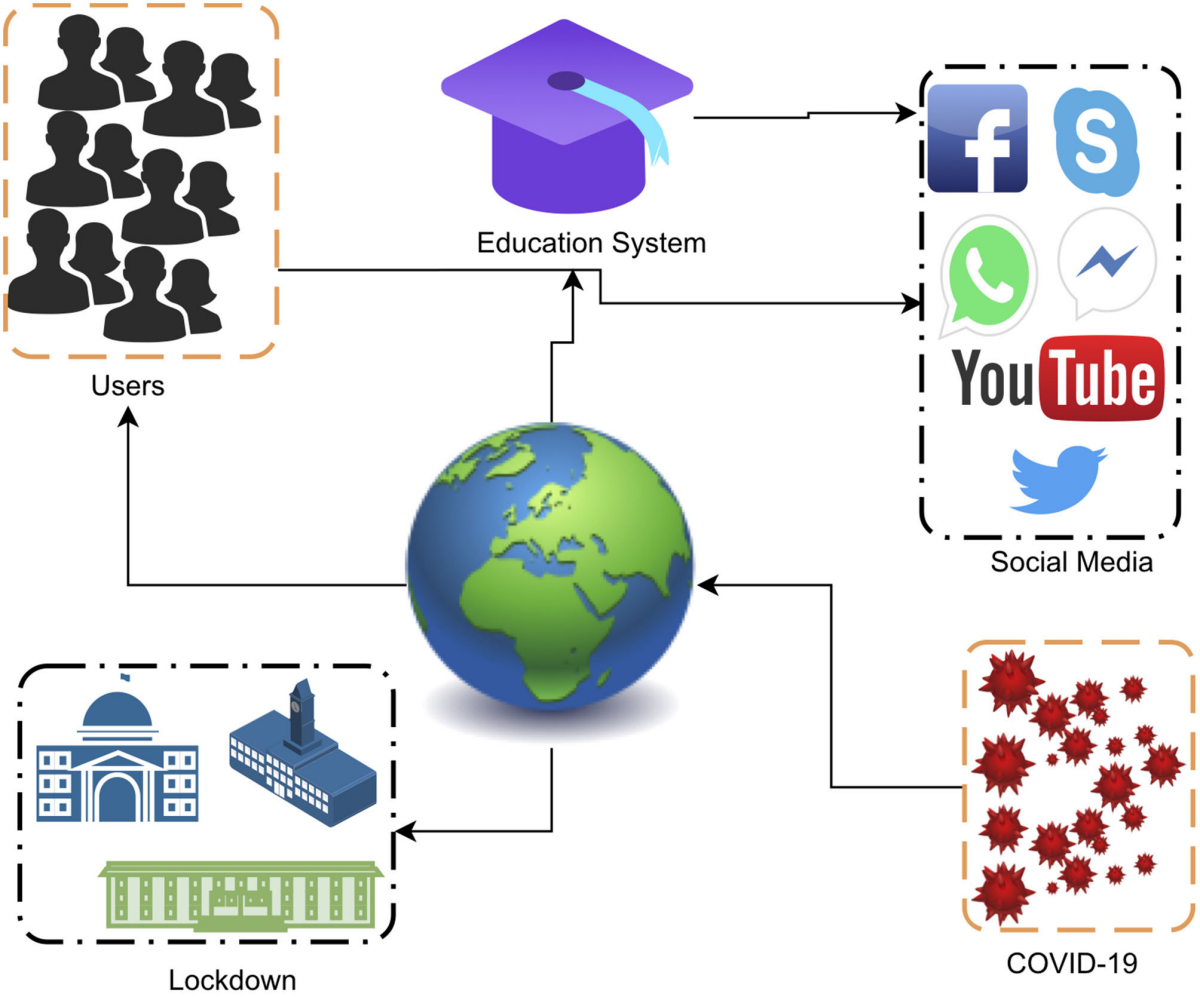
Social media adoption during the COVID-19 pandemic.

Most Pakistani people are increasingly using SM for communication, work, educational purposes, and entertainment. According to the Pakistan Telecommunication Authority (PTA), the overall Internet traffic in the country has increased by 15%, with SM platforms accounting for the largest proportion of this increase (Ramsha, 2020). Due to the ban on public gatherings, Pakistani users want to connect and communicate on Instagram, Facebook, WhatsApp, YouTube, and Twitter, as evidenced by the increase in the number of active users on all platforms during the COVID-19 period (Khan, 2020). Moreover, during March and April 2020, Facebook usage increased from 2.8 to 6.94% in its Pakistani user base. As offices, schools, and universities become more accessible remotely, the need for messaging connectivity continues to grow. WhatsApp, the most widely used messenger service in Pakistan, saw its usage increase by 23.5% over the same period at the start of the outbreak (Ramsha, 2020). Most academic institutes provide online classes using different applications, such as Zoom, WhatsApp, and Google Classroom (Adnan, 2020). As per the PTA report, video conferencing calls increased five-fold during COVID-19. The data show that on February 26, Zoom users totaled 4,149, while on April 8, its user base reached 84,469. Likewise, the number of Zoom application users increased from 5,404 to over 23,000 over 44 days (Ramsha, 2020). Figure 1 shows the impact of COVID-19 on global education systems. The traditional education system has been tremendously impacted by social distancing and other health-related measures adopted worldwide due to the COVID-19 pandemic. However, online education systems have supported the continuance of education using the already available Internet technology. In this scenario, SM platforms have provided an extensive learning platform to involve individuals in the discussion process related to learning topics among peers and student-teacher interactions. Moreover, SM platforms have helped in learning further topics in the related areas of study. Social media platforms provide an alternative to face-to-face discussions, extensively supporting students worldwide.

During the COVID-19 pandemic, multiple studies have been conducted in various research directions, including SM misinformation (Islam et al., 2020), knowledge dissemination (Chan et al., 2020), social distancing (Greenhow and Chapman, 2020), shaping knowledge (Karasneh et al., 2021), business education (Krishnamurthy, 2020), and games (Laato et al., 2020). In contrast, research on SM adoption for collaborative learning (CL) purposes by students facing the ongoing COVID-19 constraints is limited. Therefore, this study's main emphasis lies in the student community adopting the use of SM platforms for CL. Considering the literature gap identified, our study's main objectives were as follows:

- To investigate the factors that trigger SM adoption by the student community during COVID-19.
- To investigate the impact of using social media-based CL on students' learning performance.
- To investigate how students during COVID-19 collaborate with peers by using SM. Based on the study objectives, this study can open up new opportunities to integrate SM into progressive education and to leverage the exciting benefits of CL tools. We investigated how CL and perceived enjoyment (PE) may motivate individuals' SM adoption and how users' technology-related beliefs, specifically perceived ease of use (PEU) and perceived usefulness (PU) of SM, may intervene in the relationship between these factors. The target population was narrowed down to the Pakistani public university student community. Based on the technology acceptance model (TAM) by Davis (1989) and constructivism theory (CT) by Vygotsky (1978), this study contributes to the literature by conceptualizing and empirically testing a student SM adoption model.

## Literature Review

### Social Media for Learning

COVID-19 was first identified on December 8, 2019, in Wuhan (Hubei Province, China) (Khan et al., 2020). First, it began to spread in China and soon spread globally. UNESCO reported that the closure of academic institutes caused by the pandemic has affected 890 million students in 114 countries. Online learning has become a new routine for some students; however, it has brought huge challenges. According to Almaiah et al. (2020) online learning does not only involve infrastructure issues but also some other issues such as online learning system technical issues, change management issues, course design issues, computer self-efficacy, and financial support issues. Given the social inequalities in many countries, not all students have access to this kind of education (UNESCO, 2020). More than 144 countries closed academic institutions, affecting more than 67.7% of students worldwide. Some other countries closed academic institutions at the regional level, and if these closures occur at the national level, millions of other students will suffer disruption in their education (UNESCO, 2020). The social distance phenomenon has led to many rapid changes in the higher education landscape. Historically, the collective student community has never suddenly shifted from face-to-face instruction to distant instruction using digital technologies. Academic institutions have been compelled to adopt new ways to keep operating and maintain communication using digital technology, and to entirely restructure their education models in responding to pandemic-specific demand criteria. Digital technologies are increasingly used in business and educational sectors, and, on a societal level, increased digital technology use enables people worldwide to keep in touch (Dwivedi et al., 2020).

In the educational context, the adoption level of emerging web technologies is rapidly increasing. The extensive popularity of SM in education makes it essential for teachers and students to understand and adopt SM sites to establish future educational strategies and deploy present course materials on emerging technology-powered platforms (Sarwar et al., 2019; Bai et al., 2021). The use of the Internet for social networking is prevalent among youth. Collaborative technology contributes to an online community that can interact rapidly and easily. SM adoption in education has been investigated in several contexts. However, in the context of higher education only one study, which was a small-scale study, has closely observed students' adoption of SM for e-learning (Gunasagaran et al., 2019). During a pandemic, higher education institutions can use e-learning systems to help manage, plan, deliver, and track students' academic learning and faculty teaching activities (Almaiah et al., 2020). Furthermore, SM promote collaboration and participation among students and improve their learning performance (Al-Rahmi et al., 2020). Increased SM assimilation and use has become an important prerequisite for different learning applications and other related resources. Such aspects are considered useful because SM tools work through Internet-based mechanisms to disseminate and exchange information and create an environment for collaboration (Esam and Hashim, 2016).

Besides, the unprecedented rise of mobile technology in recent years has positively impacted the interaction and collaboration between teachers and students. SM provide unique features, such as CL, open-loop feedback, and two-way communication. These features enable many people to easily share their ideas, opinions, experiences, prospects, information, and knowledge freely via SM (Rau et al., 2008; Al-Rahmi et al., 2018). Moreover, SM usage enhances students' communication skills, and this means of communication enables them to collaborate and communicate despite geographical constraints, boosting their learning performance (Williams et al., 2012; Qi, 2019; Berkani, 2020) and encouraging them to work in groups; thus, members help each other by correcting each other's errors, improving their learning progress or performance (Paul et al., 2005). SM provides a platform for students and teachers to discuss their concepts and examine them with their peers (Tess, 2013). It is also a suitable way for students to easily receive feedback from their peers (Rahman et al., 2019). Considering all these advantages, we believe that SM is a valuable educational tool that can be used to enrich learning behavior (Al-Bahrani et al., 2015).

### Technology Adoption Models

Technology acceptance among its users is vital to ensuring the success of the system's implementation. Hence, it is important to understand and identify the factors that affect students' acceptance of SM learning. Scholars have presented several theories to determine the important factors that contribute to the acceptance of technology and SM in teaching and learning. Among these theories, the TAM, developed by Davis (1989), has been widely used in studies aiming to determine the factors affecting users' acceptance of new technology (Almaiah et al., 2016). The TAM model focuses on two primary factors, namely PEU and PU, which influence individuals' intention to use new technology. According to the TAM model, external variables influence individuals' internal beliefs, and the sequential relationship between individuals' personal beliefs, attitudes, and behavioral intentions leads them to use the actual system, which, in turn, helps researchers predict the acceptance of technology by its users. Many studies have used the TAM to explore the acceptance of technology among students in its original form (Davis, 1989), while others have used the extended model (Almaiah et al., 2016).

In addition to the TAM, other theories such as the Theory of Reasoned Action (TRA), the Innovation Diffusion Theory (IDT), DeLone and McLean's Information System Success Model (DL&ML model), and the Unified Theory of Acceptance and Use of Technology (UTAUT) are also used to investigate dominant determinants of accepting technology in learning environments. The TRA, developed by Fishbein and Ajzen (1975), suggests that individuals' behavior is determined by their intention to perform the behavior. This theory holds that one's intention to engage in a specific behavior is the best predictor of his/her engagement in that behavior. Regarding the IDT, Rogers proposed a diffusion process in which innovation is transferred and adopted within certain social systems over time, involving four basic elements: innovation, communication channel, time, and the social system. This diffusion results in the adoption of new ideas and behaviors among individuals who are part of the social system. However, for adoption to occur, individuals must perceive the behavior as new or innovative. The DL&ML model, developed by DeLone and McLean (2003), offers a broad assessment of the extent of information system success, which involves six components of which three, namely, service quality, system quality, and information quality, affect user satisfaction and intention to use, which, in turn, result in net benefits. Aside from these models, Venkatesh et al. (2003) proposed the UTAUT model by combining usage models to explain individuals' acceptance of an information technology and intention to use the same.

All these models and theories have been used, modified, and confirmed by prior research examining technology acceptance. Almaiah et al. (2016) employed the TAM model in the Jordanian context to examine students' acceptance of smart technology in learning. This study found that external quality factors positively affect students' adoption of new technology. Almaiah and Alismaiel (2018) combined the TAM with the DL&ML model to examine the effect of quality factors on the acceptance of mobile learning applications and found that the content, service, and quality of the system encourage students to use learning applications. Al-Shihi et al. (2018) combined the TAM and UTAUT models to explore the determinants of mobile learning acceptance in Oman. Cheng (2012) conducted a study in Taiwan to determine the factors influencing mobile learning acceptance by combining the TAM with the IDT. Similarly, Alamri et al. (2020) employed the TAM to explore the impact of SM applications on students' achievements in education sustainability in higher education. Collectively, these cited studies suggest that the TAM is a valuable and beneficial framework for explaining the use of technology and the Internet in educational settings.

## Theoretical Background and Hypotheses

**Figure 2:**
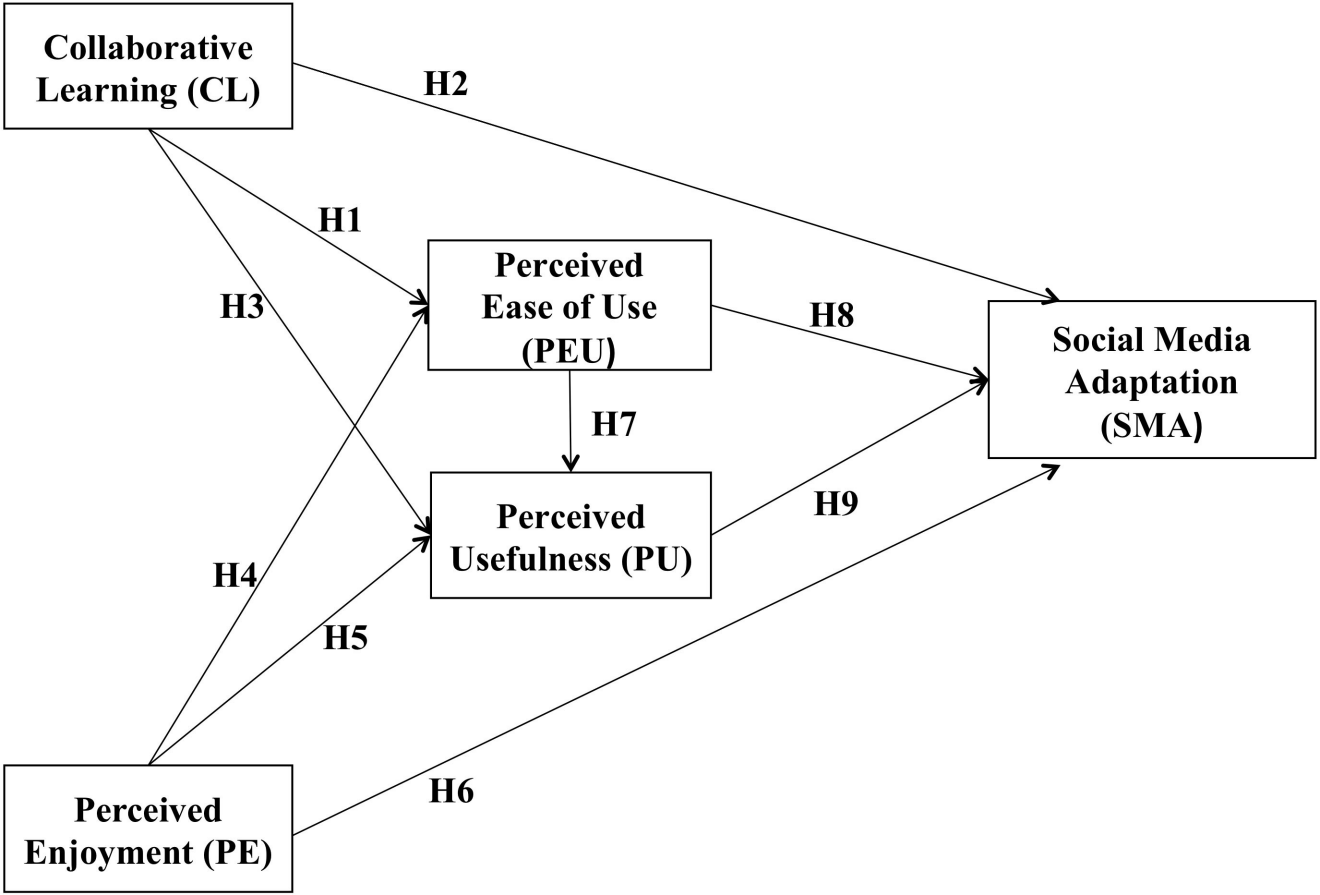
Conceptual model based on TAM and CT.

In this study, we developed a theoretical framework by merging two theories, TAM and CT. Davis designed the TAM to establish the causal relationship between the internal views, perspective, and users' intentions to adopt computer technology (Davis, 1989). Researchers have widely used the TAM to study computer technology and information systems. For example, Alamri et al. (2020) used the TAM to examine the impact of SM applications on students' achievement in higher education in Saudi Arabia. They combined the key aspects of the constructivist learning approach with TAM to find that SM applications positively affect students' satisfaction and academic performance. Chandra applied TAM to investigate users' adoption of online auctions (Chandra, 2015). Moreover, CT purports that learning is a continuous and life-long process, resulting from acting in situations (Brown et al., 1989). Students learn by collaborating and working together as peers, applying their comprehensive knowledge to solve problems (Tam, 2000). From a constructivist perspective, learning is mutually constructed through cooperation and communication with others. In the same way, based on CT activities, people acquire knowledge through communication with peers (Golub, 1988). Figure 2 shows the conceptual model used in this study.

### Collaborative Learning

CL refers to how students interact via SM with peers, colleagues, friends, and teachers to communicate for CL purposes. The means of communication and CL environments have changed due to SM platforms. CL is considered an important instructional technique to overcome the communication gap among students (Bernard and Rubalcava, 2000). Students who engage in common interests, including socializing with each other, tend to use different SM sites (Arteaga Sánchez et al., 2014; Sobaih et al., 2016). SM also plays an important role in community development, enhancing collaboration, and communication between community members (Arteaga Sánchez et al., 2014). Prior studies demonstrate that SM sites are useful for university and college students for educational purposes (Forkosh-Baruch and Hershkovitz, 2012). Group interaction is positively influenced by CL via SM and participating in social networking, where individuals gain diverse skills through engagement and improved performance (Mazer et al., 2007; Liu et al., 2011; Ku et al., 2013). SM significantly influence students' academic performance, and it is believed that Facebook is suitable for interacting with classmates and teachers (Cao and Hong, 2011; Lin et al., 2019). In terms of education, the utilization of SM promotes CL among students (Liao et al., 2015; Alamri et al., 2020). In particular, many researchers have emphasized the significance of CL and highlighted its potential for enhancing academic performance and empowerment. This is possible if students' needs and their formative assessment are prioritized, and a community classroom is created that promotes student participation, improves their academic performance, and manages the exchange of knowledge among them. From this perspective, SM is highly useful for creating academic groups to improve students' academic performance (Pulido et al., 2020). SM enhances students' learning activities and promotes group communication, so their utilization as learning and teaching tools by educational institutions should be encouraged (Merle and Freberg, 2016). Moreover, social networks enable teachers to deliver information, interact with students, and offer students various teaching methods, encouraging them to become active students (Merle and Freberg, 2016). Sugimoto et al. (2015) argue that these enriched learning experiences also help increase students' participation in the classroom. Utilizing social networking sites in education has not only enhanced the learning process itself, but has also enhanced students' psychological health, social interaction, and skills. Universities and higher education institutions should focus on delivering awareness sessions as a way to encourage students and faculty to use mobile SM apps to maximize learning outcomes (Almaiah et al., 2020). Because of their inherent utility and ease of use, students often utilize information and communications technologies, particularly SM, to better collaborate with peers (Wang, 2010; Koh and Lim, 2012). Based on the above discussion, the following hypotheses were developed:

**H1**: CL is positively related to PEU.
**H2**: CL is positively related to SM adoption.
**H3**: CL is positively related to PU.

### Perceived Enjoyment

PE refers to the degree to which an information technology system serves as an antecedent of perceived user-friendliness and PU. Our study proposes that enjoying technology, specifically enjoying SM, is conducive to the perception of PEU and is perceived as useful for CL. PE pertains to the intrinsic motivation or the affective element that affects the PEU and user acceptance constructs of the TAM (Van der Heijden et al., 2003; Venkatesh and Bala, 2008). PE is defined as the degree to which the service provided by the learning management systems is considered to be enjoyable *per se*, excluding any performance concern regarding the use of the system (Venkatesh, 2000; Van der Heijden, 2004). Scholars have reported adopting new technologies as performance-enhancing devices and pleasure sources (Venkatesh, 2000; Koenig-Lewis et al., 2015). Furthermore, Van der Heijden (2004) and Agarwal and Karahanna (2000) suggest that PE can be a precursor to PEU and PU, showing that pleasant technology is also considered easier to use and more useful. Based on the above literature, we proposed the following hypotheses:

**H4**: PE is positively related to PEU.
**H5**: PE is positively related to PU.
**H6**: PE is positively related to SM adoption.

### Perceived Ease of Use and Usefulness

PEU refers to the degree to which a user believes that using a specific information technology system will be simple and comparatively free of physical or mental effort (Davis, 1989). In the context of SM, PEU refers to the degree to which SM sites are easy to use (Carlos Martins Rodrigues Pinho and Soares, 2011; Rauniar et al., 2014). PEU and PU are reliable predictors of the adoption rate of different information technologies, such as mobile learning and Internet-based learning systems (Saadé and Bahli, 2005; Althunibat, 2015). Bhattacherjee (2001) stated that PU is the user's perception of the benefits of using a technology system, while Davis (1989) explains PU as subjective, optimistic ideas about the potential benefits of a certain technology system that arise after its use. This research expands on these definitions by defining PU as a positive subjective notion held by users that the adoption of SM would enhance CL. Based on the above discussion, the following hypotheses were proposed:

**H7**: PEU is positively related to PU.
**H8**: PEU is positively related to SM adoption.
**H9**: PU is positively related SM adoption.

## Materials and Methods

We decided to study the driving factors behind SM adoption of by public university students in Pakistan who experienced interruption in regular CL processes during the COVID-19 period from July to August.

### Measurement Development

Due to geographical constraints, for faster distribution, and to minimize the issue of invalid or missing data, an online survey method was used to collect primary data from those who were expected to display excessive SM usage patterns (Luo and Chea, 2020) and who widely adopt SM (Arshad and Akram, 2018). To research SM adoption, five constructs are measured using a five-point Likert scale ranging from 1 (Strongly Agree) to 5 (Strongly Disagree). These constructs include PU, PEU (Alenazy et al., 2019), CL, PE (Sarwar et al., 2019), and SM adoption (Al-Rahmi et al., 2018). We developed a questionnaire in English to collect corresponding data. All participants signed a consent letter to participate in the study voluntarily. At the beginning of the survey, respondents were also provided with a concise overview of the aim of the research to increase their basic understanding. Respondents were also ensured that the data would only be used for educational purposes, so they were not asked to provide other information. A pilot study (pilot test) with 23 respondents who used SM for more than 2 h a day revealed a Cronbach's alpha value of 0.7 for each construct, providing an appropriate statistical basis for further in-depth research. Subsequently, revisions based on the feedback obtained were implemented, resulting in the formal survey.

The validity of any inference derived from data relies on the use of appropriate measurement methods. Consistent with previous literature on explanatory and confirmatory research (Henseler et al., 2016), a structural equation modeling (SEM) approach, partial least squares (PLS), was selected as the core measurement method. PLS produces exemplary consistent estimations of a composite model (Benitez et al., 2020) and has been found to have improved accuracy over covariance-based SEM (van Riel et al., 2017). Additionally, PLS enables us to circumvent the necessity of variables to follow a multivariate normal distribution (Chin et al., 2003) by performing component-based SEM. After the primary data collection, it is necessary to assess the common method bias to ensure that there is no systemic bias affecting the collected data (Podsakoff et al., 2003; Valaei et al., 2017).

### Formal Survey

The revised questionnaire was distributed via email, Facebook, WhatsApp, and WeChat over a period of 27 days. To prevent bias, no personal data other than gender and age were obtained. The tools used to conduct the analysis were Jamovi software, for demographic analysis, and SmartPLS 3 software for data analysis of our conceptual model.

### Sample Characteristics and Descriptive Statistics

The demographic characteristics of the participants are presented in Table 1. A total of 325 responses were obtained, 289 of which were deemed valid. Of these, 172 respondents were male, and 117 were female. Validity was defined as abiding by two parameters: (1) a complete observation, and (2) participants reporting a minimum use of SM of 2 h per day.

**Table 1:** Respondents' demographics.

**Demographics**	**Criteria**	**Percentage**
Gender	Male	59.5
	Female	40.4
	Bachelor's (Hons)	29.4
Qualification	Master's	42.2
	M.Phil.	18.3
	Ph.D.	10.0
Usage of social media per day	2–3 h	14.8
	3–4 h	30.7
	5 or more than 5 h	54.3

### Common Method Variance

We performed a Harman single factor test to assess the potential existence of common method variance (CMV) in our data (Podsakoff et al., 2012). The results revealed that the first-factor value was 38.3%, which was lower than the threshold value of 50%. As such, we concluded that there was no common method bias in the data, and no CMV issue existed in the data. Second, the common latent factor approach suggested by Podsakoff et al. (2012) was also employed. As per this approach, standard regression weights were first computed using a confirmatory factor analysis. Confirmatory factor analysis was then conducted by including a common latent factor in the research model. The comparison of the regression weights of both analyses revealed no dominant factor emerging from the results, meaning that the common method bias was not an issue in this study.

## Research Analysis

For this study, we performed SEM analysis to determine the consistency of the measuring tool and to compare it with the study hypotheses, we used SmartPLS 3.0 and applied the PLS method (Molinillo et al., 2018). PLS-SEM is suitable for studying technology acceptance that stresses predictive modeling (Venkatesh and Davis, 2000; Venkatesh and Bala, 2008). The results of this study are divided into two parts: the first part describes the measurement model and reports checks of the consistency between the endogenous and exogenous variables by identifying the composite reliability (CR), convergent validity (CV), Cronbach's alpha (CA), average variance extracted (AVE), factor loadings, and rho_A. The second part discusses our structural model and evaluation of the study hypotheses.

### Measurement Model

The reliability and validity results of our measurement model are listed in Table 2. The CR values of all constructs in our measurement model were >0.60, as recommended by Hair et al. (2017). The AVE value of each construct should be larger than the suggested threshold of 0.50 (Fornell and Larcker, 1981), and we observed it to be as so. Furthermore, our study's results indicated that all constructs' CA values were above the recommended value of 0.70 (Fornell and Larcker, 1981; Kannan and Tan, 2005; Lee et al., 2005; Wu et al., 2007).

**Table 2:** Construct validity.

**Constructs**	**Items**	**Loading Values**	**Cronbach's a**	**rho_A**	**CR**	**AVE**
Collaborative learning	CL1	0.873	0.883	0.885	0.919	0.740
	CL2	0.855				
	CL3	0.880				
	CL4	0.832				
Perceived enjoyment	PE1	0.863	0.797	0.814	0.880	0.709
	PE2	0.845				
	PE3	0.818				
Perceived ease of use	PEU1	0.732	0.772	0.885	0.839	0.567
	PEU2	0.821				
	PEU3	0.706				
	PEU4	0.750				
Perceived usefulness	PU1	0.812	0.810	0.880	0.867	0.620
	PU2	0.761				
	PU3	0.804				
	PU4	0.773				
Social media adoption	SMA1	0.864	0.853	0.853	0.911	0.772
	SMA2	0.879				
	SMA3	0.893				

As an alternative metric for measuring reliability, Dijkstra and Henseler (2015) proposed the rho_A. Our study results of all constructs using Dijkstra-Henseler's rho_A were all above the threshold value of 0.70, indicating construct reliability. Table 3 indicates the discriminant validity (DV) results based on the Fornell-Larcker criterion.

**Table 3:** Discriminant validity.

**Fornell-Larcker criterion**						**Heterotrait-monotrait ratio (HTMT**)					
**Constructs**	**CL**	**PE**	**PEU**	**PU**	**SMA**	**Constructs**	**CL**	**PE**	**PEU**	**PU**	**SMA**
CL	**0.860**					CL					
PE	0.278	**0.842**				PE	0.329				
PEU	0.415	0.489	**0.753**			PEU	0.430	0.551			
PU	0.431	0.428	0.616	**0.788**		PU	0.453	0.469	0.641		
SMA	0.538	0.486	0.707	0.765	**0.879**	SMA	0.619	0.580	0.730	0.827	

*Square root of the AVE in bold in the main diagonal*.

As shown in bold, the values of the square root of the AVE on the diagonals are greater than the correlations among the constructs. This shows that the constructs are strongly related to their respective indicators compared to other models (Fornell and Larcker, 1981), indicating better DV (Hair et al., 2016). Furthermore, as an alternative, Dijkstra and Henseler proposed the heterotrait-monotrait ratio of correlations (HTMT), where values smaller than 0.85 or 0.90 reliably distinguish latent variable validity (Dijkstra and Henseler, 2015). In our study, all values met significant thresholds, as shown in Table 3. In summary, the measurement model showed appropriate reliability, CV, and DV in our conceptual model.

### Structural Model Assessment

Previous literature suggests assessing the structural model by looking at the beta value (β-value), the *R*^2^-value, and the corresponding t values obtained via a bootstrapping procedure with 5,000 resamples (Hair et al., 2016). Furthermore, it is recommended to report the effect sizes (f^2^) and predictive relevance (*Q*^2^), the results of which are shown in Table 4. *f* ^2^ > 0, 0.15, and 0.35 indicate a small, medium, and large effect size, respectively, and *Q*^2^ > 0, 0.25, and 0.50 indicate small, medium, and large predictive relevance, respectively (Hair et al., 2019).

**Table 4:** Standard assessment criteria of a structure model.

**Constructs**		***f^**2**^***		***R*^**2**^**	***R*^**2**^ Adjusted**	***Q*^**2**^**
CL	0.125	0.057	0.097			
PE	0.224	0.028	0.020			
PEU		0.255	0.168	0.323	0.319	0.152
PU			0.413	0.433	0.427	0.234
SMA				0.710	0.706	0.542

As Sullivan and Feinn (2012) argue, the *p*-value determines whether the effect exists, but it does not reveal the size of the effect. The collinearity values were evaluated by producing the variance inflation factor (VIF). VIF values higher than 5 indicate collinearity. The recovered VIF values were entirely within acceptable range values, that is, <5 (Mason and Perreault, 1991; Becker et al., 2014), as shown in Table 5.

**Table 5:** Standard assessment criteria of a structure model.

**Constructs**	**VIF**
CL1	2.683
CL2	2.479
CL3	2.447
CL4	1.966
PE1	1.636
PE2	1.854
PE3	1.662
PEU1	1.678
PEU2	1.236
PEU3	1.674
PEU4	1.729
PU1	1.344
PU2	2.059
PU3	1.850
PU4	2.046
SMA1	1.898
SMA2	2.191
SMA3	2.339

**Figure 3:**
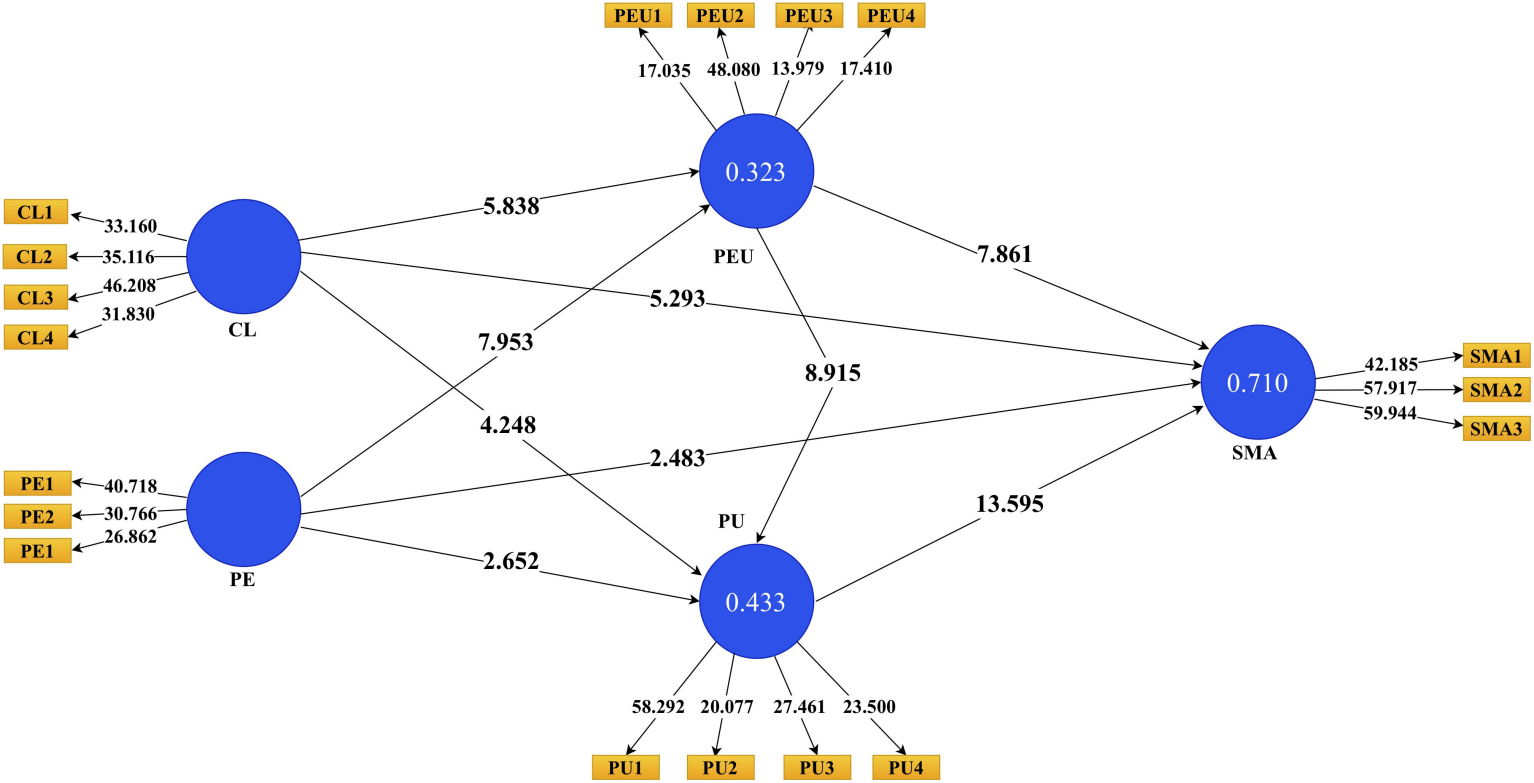
Bootstrapping path coefficient results.

From the results, we conclude that collinearity did not exist in our data. The results of the structural model assessment are shown in Figure 3 and Table 6. Our study hypothesis results showed that CL (β = 0.302, *t* = 5.838, *p* < 0.001) and PE (β = 0.405, *t* = 7.953, *p* < 0.001) had a significant relationship with PEU. This result supports H3 and H5. Additionally, CL was found to be significantly associated with SM adoption (β = 0.191, *t* = 5.293, *p* < 0.001), which also provides grounds for accepting H6. Moreover, PEU and PU (β = 0.462, *t* = 8.915, *p* < 0.001) were positively and significantly associated. Finally, the H8 (β = 0.301, *t* = 7.861, *p* < 0.001) and H9 (β = 0.460, *t* 13.595, *p* < 0.001) results revealed that the relationship of PEU and PU with SM adoption was positive and significant. Overall, the results supported all hypotheses.

**Table 6:** Path coefficient results.

**No**		**OS (O)**	***M***	***SD***	***T*-Value**	***P*-Values**	**Result**
1	CL -> PEU	0.302	0.303	0.052	5.838	0.000	Accepted
2	CL -> SMA	0.191	0.191	0.036	5.293	0.000	Accepted
3	CL -> PU	0.199	0.201	0.047	4.248	0.000	Accepted
4	PE -> PEU	0.405	0.407	0.051	7.953	0.000	Accepted
5	PE -> PU	0.146	0.147	0.055	2.652	0.008	Accepted
6	PE -> SMA	0.089	0.090	0.036	2.483	0.013	Accepted
7	PEU -> PU	0.462	0.462	0.052	8.915	0.000	Accepted
8	PEU -> SMA	0.301	0.303	0.038	7.861	0.000	Accepted
9	PU -> SMA	0.460	0.456	0.034	13.595	0.000	Accepted

## Discussion

From its onset until December 31, 2020, over 81 million new COVID-19 cases were reported by the World Health Organization (2021). The ongoing COVID-19 pandemic has disrupted every facet of life, including healthcare, education, and the economy (Li et al., 2020). Educational institutes were shut down, and students globally were confined to staying at home. As yet, it is uncertain when the imposed lockdowns will be lifted. Social distancing has led to increased SM use among students. SM is a convenient way of communication, and it enables students and teachers to connect and practice social distancing more effectively (Vordos et al., 2020).

This study investigates the basic motivation behind the adoption of SM for CL in the student community during COVID-19. To achieve the aim of this study, we proposed a conceptual framework that expands on the TAM and CT. In the present model, we defined an SM feature, CL, as the key variable underlying SM adoption and identified PEU and PU as important contributing variables. For the proposed hypotheses testing, we collected data by surveying students. The results suggest that CL plays a key role in determining the user's verdict to adopt SM. Moreover, the PEU and PU of SM play a mediating role in the relationship between SM characteristics and adoption. Overall, the study's findings strongly support the adoption of SM for educational purposes. This notion is strengthened by the alignment, which is consistent with results reported in previous studies (Sobaih et al., 2016; Berger, 2017).

Online learning is becoming more mainstream, and discussions are ripe between researchers about the utility of this medium given the breakthroughs in the ease of use of SM for academic communication. In addition, the study is well-placed to strengthen and advance the literature on CL, PE, and its role in SM adoption by students (Mazman and Usluel, 2010; Arteaga Sánchez et al., 2014).

The study results support the notion that the SM feature CL is a predictor of SM adoption by students in Pakistan. This reveals that students considered that SM enables CL, enhancing communication between friends and classmates. Besides, this result is consistent with that of a previous study by Arshad and Akram (2018). Our study also shows that students engage in various SM sites where they discuss their educational issues and collaborate with others, which positively impacts their learning performance. Furthermore, the results imply that SM facilitates students to become more creative and dynamic and enables them to easily collaborate with instructors. The study results are supported by a previous study (Ansari and Khan, 2020).

Our findings demonstrate that CL significantly affects PU, PEU, and SM. Students in higher education who adopt SM tend to consider that this platform is useful for collaborating with others, which is consistent with the CT of learning. This result aligns with that of previous studies by Ebner (2009) and Arshad and Akram (2018), who found that SM sites such as blogs are useful for CL between students and tutors. Furthermore, PE has a significant effect on PU, PEU, and SM. This finding demonstrates that the extensive popularity of SM among students is partly due to its ease of use. Students use SM for different purposes, such as enjoyment, knowledge sharing, and CL; this result is consistent with previous literature (Koenig-Lewis et al., 2015; Al-Rahmi et al., 2020). Similarly, our study results indicate that PU and PEU also have a positive relationship with SM adoption. SM tools facilitate resource sharing, as learners perceive this medium to be easy to use and useful because it helps them share information with the relevant person more effectively and efficiently. This result echoes previous research (Al-Rahmi et al., 2018; Sarwar et al., 2019). In addition, in this study, PEU was shown to have a significant relationship with PU, which was also reported by Alenazy et al. (2019).

SM can change the conventional educational method and provide a platform for students where they can directly communicate and collaborate with different people globally (Reid and Ostashewski, 2010; Forkosh-Baruch and Hershkovitz, 2012). This point is supported by two theoretical perspectives: the CT and computer-mediated learning (CML). The emphasis of CT on social contact and collaboration lies in the fact that CML eradicates topographical hurdles. Hence, to gain useful learning experiences related to CL, it is imperative to develop social groups to exercise and use CL setting abilities on SM networks.

## Research Implications

The present study has important implications for students, higher educational institutions, and policymakers. The relationship between the use of SM and its positive impact on students' performance is crucial to understanding the role of SM during a pandemic. The findings are relevant to those interested in enhancing online learning or the SM tools utilized for CL. This study furthers our understanding of why students choose to adopt SM sites during COVID-19. Through a greater comprehension of intention determinants, inclination toward e-learning among students, and useful technology, informed policy decisions can be reached for educational technology implementation in tertiary education institutions. The findings of this study will increase educational administrators' awareness of the benefits of advanced technology, such as SM in academic institutions, and assist them in developing an interesting and suitable online learning environment for the student community. Furthermore, teachers and students should consider SM as an informal learning tool that creates a comfortable environment for CL and social interaction. Educational administrators, policymakers, and teachers can use SM as a complementary learning tool, and students can use it for CL. Further, based on our study results, we suggest that educational institutions develop their own pages and groups on different SM platforms and invite students to join these groups and pages, which may help students tackle educational problems. Students can join groups or pages using official email addresses. Such initiatives may reduce student search effort, a constraint, and thus more efficiently achieve favorable CL with peers regardless of location or time.

## Limitations and Future Work

The present study identified some interesting findings; nevertheless, several limitations exist. Notably, the sample population was limited to public universities in Pakistan. Hence, this study's results may not conclusively reflect private university students' tendency toward SM adoption during COVID-19 in Pakistan. Additionally, this study did not distinguish between specific SM platforms such as Facebook, Instagram, and Twitter. As such, it did not yield detailed platform-specific descriptive statistics for use during COVID-19 for educational purposes or collaboration. Accordingly, future research should be confined to a specific SM platform. Future research should be conducted with faculty members to understand their perspective on SM adoption for CL during the pandemic. Although the model has been tested in Pakistan, future research should replicate or extend the proposed conceptual model in technologically advanced countries with different economic and cultural conditions.

## Conclusion

This study advances research on SM adoption by students during COVID-19 for the purpose of CL. This is achieved by proposing and empirically testing a conceptual model based on the TAM and CT. Social media, as a tool, as well as its features, is indispensable and extremely vital for students in higher education. Concurrently, SM may be useful in enhancing learning performance, knowledge sharing, and collaboration among students. Social media provides a learning platform for students where they can easily communicate with their peers, teachers, and subject specialists. Furthermore, the use of SM is conducive to enhancing learners' CL. Particularly in a time of growing focus on expedient delivery of coursework through digital technologies, students, higher educational institutions, and policymakers may ascertain a positive impact on CL through SM adoption by students. The study results indicate that CL, PE, PEU, and PU are vital contributors of SM adoption by students in higher education. With an extended understanding of the determinants of SM adoption motivation and inclination toward e-learning between students who experience constraints during a pandemic, informed policy decisions can be reached. Crucially, however, scope restrictions, in particular, the small sample of the study, comprising 289 public university students, and the geographic restriction of Pakistan, impede the ability to draw conclusive inferences on the effectiveness of the conceptual model; thus, replicative research in technologically advanced countries with different economic and cultural conditions is warranted.

## Data Availability Statement

The data analyzed in this study is subject to the following licenses/restrictions: data is available on request. Requests to access these datasets should be directed to azeem20037@gmail.com.

## Ethics Statement

The studies involving human participants were reviewed and approved by Hunan University, Nanjing University. The patients/participants provided their written informed consent to participate in this study.

## Author Contributions

MNK and MAA: conceptualization and formal analysis. MNK and DS: methodology. MNK: software. MNK, MAA, and DS: resources and writing—original draft preparation. MNK, MAA, KUK, and RAL: writing—review and editing. All authors have read and agreed to the published version of the manuscript.

## Conflict of Interest

The authors declare that the research was conducted in the absence of any commercial or financial relationships that could be construed as a potential conflict of interest.
